# ADSC-derived mitochondrial nanovesicles transplantation alleviates capsular fibrosis and inflammation and improves joint mobility in a rat model of adhesive capsulitis

**DOI:** 10.1016/j.jot.2026.101195

**Published:** 2026-08-22

**Authors:** Yiming Zhu, Zeyu Wang, Ziqi Huo, Dan Zhang, Yang Zhao, Jianhao Xie, Hao Li, Tianyuan Zhao, Pinxue Li, Zhi Wang, Chunyan Jiang, Xu Li

**Affiliations:** a Beijing Jishuitan Hospital, Capital Medical University, Beijing, China; b Beijing Key Lab of Regenerative Medicine in Orthopedics, Key Laboratory of Musculoskeletal Trauma & War Injuries PLA, Institute of Orthopedics, The First Medical Center, Chinese PLA General Hospital, Beijing, China; c Department of Orthopedics, Peking University Third Hospital, 49 Huayuan North Road, Beijing, 100191, China

**Keywords:** Adhesive capsulitis, Adipose-derived stem cell, Capsular fibrosis, Joint inflammation, Mitochondrial dysfunction, Mitochondrial transplantation

## Abstract

**Background:**

Adhesive capsulitis (frozen shoulder) is a prevalent condition characterized by shoulder pain and progressive motion loss. Mitochondrial metabolic dysregulation is an underlying driver of chronic inflammation and fibrosis. This study aimed to characterize mitochondrial metabolic abnormalities in patient capsular tissue and evaluate a therapy using adipose-derived stem cell (ADSC) derived mitochondrial nanovesicles transplantation.

**Methods:**

Single-cell RNA sequencing was utilized to analyze the expression of nuclear-encoded genes related to mitochondrial metabolism in fibroblast subpopulations from human adhesive capsulitis capsular tissue. ADSC-derived membranes were extruded together with exogenous mitochondria to generate engineered mitochondrial nanovesicles (AD-Mito-NPs). An inflammatory fibroblast model was employed to assess the uptake of AD-Mito-NPs, along with associated transcriptomic and metabolomic changes, and their effects on apoptosis, inflammation, and extracellular matrix (ECM) remodeling. Finally, AD-Mito-NPs were locally injected into a rat model to evaluate joint movement and histopathology.

**Results:**

AD-Mito-NPs retained intact respiratory function, high fibroblast internalization efficiency, and stable physicochemical properties for up to 7 days. *In vitro* inflammatory models verified that AD-Mito-NPs reversed IL-1β-triggered mitochondrial injury and strengthened mitochondrial oxidative phosphorylation. Furthermore, AD-Mito-NPs alleviated intracellular reactive oxygen species accumulation and fibroblast apoptosis, mitigated inflammatory responses, and remodeled extracellular matrix homeostasis. *In vivo*, intra-articular administration of AD-Mito-NPs improved shoulder joint mobility, attenuated capsular thickening and disordered collagen arrangement, and suppressed local inflammation in a rat model of adhesive capsulitis.

**Conclusion:**

Mitochondrial metabolic imbalance is a factor driving capsular fibrosis in adhesive capsulitis. Engineered mitochondrial transplantation offers therapeutic benefits by enhancing mitochondrial energy production, mitigating oxidative stress and inflammation, and restoring ECM balance.

**The translational potential of this article:**

This article identifies mitochondrial metabolic dysregulation as a key driver of adhesive capsulitis-related capsular fibrosis and demonstrates that engineered AD-Mito-NPs are a safe platform for clinical translation. These NPs effectively enhance energy metabolism, reduce inflammation, and improve shoulder mobility in models, providing a promising alternative to existing treatments.

## Graphical abstract

**Figure:**
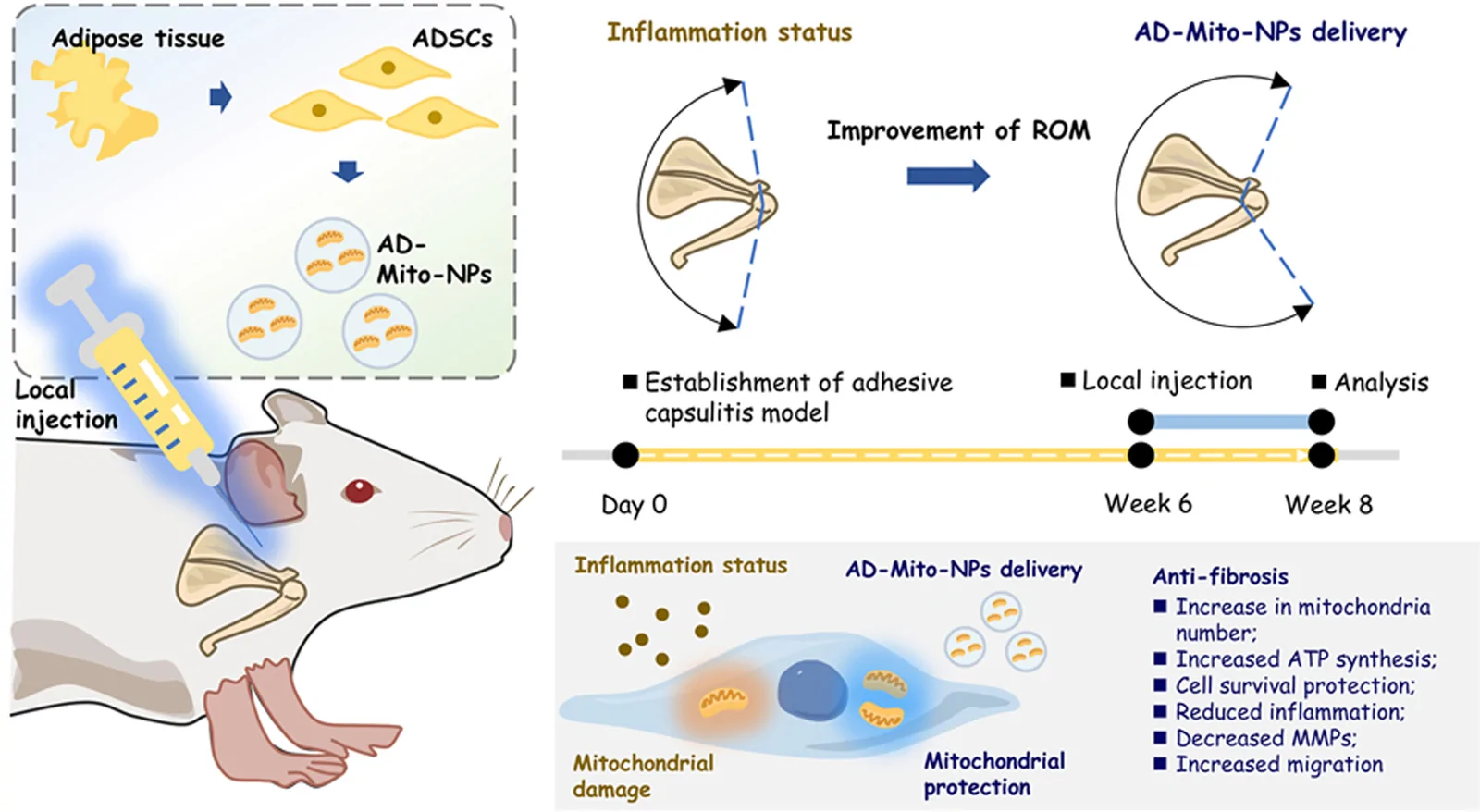

## 1 Introduction

Adhesive capsulitis, commonly called frozen shoulder, is a condition characterized by shoulder pain and limited mobility, affecting approximately 2% to 5% of the total population [[1], [2], [3]]. The main pathological features of this disease include inflammation and fibroproliferative fibrosis, and its course is typically divided into three phases: the mobility loss stage (inflammation/freezing phase, stage I), stiffness (frozen phase, stage II), and symptom resolution (thawing phase, stage III) [4]. The infiltration of immune cells (such as T lymphocytes) and the release of inflammatory mediators (such as Interleukin-17A (IL17A) and IL-6) can promote the process of fibrosis [5,6]. Fibroblasts transform into myofibroblasts and produce large amounts of type I and type III collagen, which leads to the degradation, remodeling, and imbalance of the extracellular matrix (ECM). It is often accompanied by neovascularization and abnormal innervation, eventually leading to fibrosis and contracture of the joint capsule, as well as clinical stiffness [4,7].

Although adhesive capsulitis is often considered to be a self-limiting disease with a duration of approximately 1 to 2 years, the pain and stiffness during this period severely affect the patient's quality of life [4]. In 20% to 50% of patients, pain persists for 2 to 20 years or more after follow-up [[8], [9], [10]]. The clinical treatment options for adhesive capsulitis include physical therapy, steroid injections, anti-inflammatory drugs, hyaluronic acid injections, and surgical intervention; however, their effectiveness remains unclear [4]. Novel therapeutic strategies are gradually being translated from the laboratory to the clinic to develop biologic therapies that target the inflammation-fibrosis cascade [11,12].

Aberrant fibrosis markedly impairs musculoskeletal homeostasis and is accompanied by chronic inflammation, among which mitochondrial dysfunction is a key mechanism driving the pathological progression of chronic inflammatory diseases [[13], [14], [15], [16], [17]]. In muscle and tendon tissue, impairment of energy supply and excessive reactive oxygen species (ROS) production collectively worsen the disease and are closely linked to disuse muscular atrophy [18,19]. Peroxisome proliferator-activated receptor-γ (PPAR-γ) plays a central regulatory role in mitochondrial function and turnover, energy metabolism, antioxidant defense, immune regulation, and fatty acid oxidation [20]. Its ligands can inhibit NF-κB, AP-1, and JAK/STAT signaling pathways, participating in anti-inflammatory and immune regulation, as well as regulating glucose and lipid metabolism [20]. Studies indicate that PPARγ agonists delay disease progression in a rat model of adhesive capsulitis, suggesting that targeting mitochondrial metabolism could be a potential therapeutic approach [12]. Recently, mitochondrial transfer, an emerging therapeutic technique, has shown significant promise in promoting tissue repair [21,22]. This method aims to repair or replace damaged mitochondrial networks in cells by exogenously supplying fully functional mitochondria with regular respiratory activity. The process involves isolating highly active mitochondria from healthy tissue and delivering them to diseased sites within the target tissue through local or systemic injection [23,24]. Its primary mechanisms include enhancing energy metabolism, reducing inflammatory responses, and promoting anti-apoptotic effects [25,26]. However, whether this method can modulate inflammation and inhibit fibrosis in adhesive capsulitis remains uncertain.

This study introduces engineered mitochondrial nanoparticles transfer method using adipose-derived stem cells (ADSCs) for the treatment of adhesive capsulitis. ADSCs can be easily obtained through liposuction and exhibit low immunogenicity, along with paracrine repair functions [27,28], making them a convenient source of mitochondria for transplantation therapy. ADSC membranes might help maintain the mitochondrial membrane potential. We hypothesize that engineered ADSC mitochondrial nanoparticles (AD-Mito-NPs) could restore cellular energy balance and reduce joint capsule fibrosis. This study aims to assess the therapeutic potential of mitochondrial transplantation in a rat model of adhesive capsulitis and identify relevant pathological targets.

## 2 Materials and methods

### 2.1 Single-cell sequencing data analysis

Single-cell RNA-seq profiles of shoulder capsules were downloaded from GEO (ERP143358) [29]. The dataset included four adhesive capsulitis patients and six controls with shoulder instability. Low-quality cells were removed in the original dataset if they contained fewer than 300 or more than 6500 genes or more than 10% mitochondrial reads. The remaining count matrix was normalized with Seurat's LogNormalize. The 3000 most variable genes were identified by VST and used to compute ten principal components. Harmony corrected batch effects across samples. Clusters were visualized with UMAP and annotated with canonical markers. AUCell quantified the activity of mitochondrial metabolism gene sets from GO and Reactome at the single-cell level [30]. For volcano plots and GSEA enrichment analyses, we adopted donor-level pseudobulk integration.

### 2.2 Human samples

All human sample collection procedures were approved by the Clinical Research Ethics Committee of the department. The enrolled patients met the following inclusion criteria: patients aged 45 to 65 years with rotator cuff injury (with or without concomitant adhesive capsulitis) who were scheduled for arthroscopic shoulder surgery and received concomitant capsular release during rotator cuff repair surgery. Patients were excluded if they had underlying diseases (e.g., diabetes mellitus and autoimmune diseases) that could affect inflammatory and fibrotic indicators, a history of prior shoulder surgery or trauma, shoulder tumors, infections or other shoulder lesions, or recent treatment with glucocorticoid injections, immunosuppressants, or other interventions that might alter the detection of inflammatory and fibrotic indicators. Discarded joint capsule specimens obtained during the capsular release procedure were collected for histological analysis.

### 2.3 Cell culture and treatment

Capsular fibroblasts were isolated as described [31]. Briefly, 4-week-old male Sprague–Dawley (SD) rats were euthanized under anesthesia. The limbs were disinfected with 75% ethanol for 10 min. Then, the knee and shoulder joint capsules were excised and minced into small pieces with fine scissors. These pieces were plated in Dulbecco's Modified Eagle Medium (DMEM, Gibco, 11965092) supplemented with 10% fetal bovine serum (FBS, BKMAMLAB, 110805008), 1% penicillin-streptomycin (Gibco, 15140122). The cultures were kept at 37 °C in a 5% CO_2_ atmosphere, refreshed every two days, and used when about 90% confluent.

For the isolation and culture of ADSCs, 4-week-old male Sprague–Dawley (SD) rats were euthanized under anesthesia and then disinfected in 75% ethanol for 10 min. Inguinal fat pads were excised, minced, and digested with 0.1% type I collagenase at 37 °C for 1 h on a shaker. To stop the digestion, serum-containing medium was added. The resulting cell suspension was filtered through a 100-mesh sieve, and the cells were plated in DMEM supplemented with 10% FBS and 1% penicillin-streptomycin. The cultures were maintained at 37 °C in a 5% CO_2_ incubator, refreshed every two days, and used when they reached approximately 90% confluence.

### 2.4 Cell membrane and mitochondrial extraction and Co-extrusion

Cell membranes were isolated from ADSCs following a previously published protocol [32]. ADSCs were resuspended in IB-1 buffer containing 0.5% (w/v) bovine serum albumin (BSA, Gibco, 11020021), 225 mM mannitol, 30 mM Tris-HCl, 0.5 mM EDTA, 75 mM sucrose, protease inhibitor (20 μL per 4 mL of buffer), and 1% (v/v) phosphatase inhibitor at pH 7.4. The cell suspension was sonicated on ice and then centrifuged at 800 × g and 4 °C for 10 min to remove intact cells and nuclei. The supernatant was further centrifuged at 10,000 × g and 4 °C for 20 min. The pellet was then subjected to ultracentrifugation at 100,000 × g and 4 °C for 60 min, followed by lyophilization. The lyophilized product was weighed and stored at −80 °C.

Mitochondria were isolated from ADSCs following the manufacturer's protocol and quantified using the bicinchoninic acid (BCA) assay (Gibco, 23227). The membrane fraction was resuspended in water at a concentration of 2 mg/mL. An equal volume of mitochondrial suspension was added and incubated for 30 min. The mixture was sequentially extruded through polycarbonate membranes with pore sizes of 1000 nm, 400 nm, and 200 nm using an Avanti mini-extruder. CM-mito nucleoids (named AD-Mito-NPs) were collected by centrifugation at 10,000 × g for 5 min and resuspended in PBS solution.

### 2.5 Characterization of AD-Mito-NPs

Particle morphology was visualized by transmission electron microscopy (TEM). For fluorescence colocalization detection, membranes were pre-labeled with Dil (Beyotime, C1415) for 15 min, and mitochondria were pre-labeled with Mito-Tracker green (Beyotime, C1048) for 30 min, before the extrusion. Free dye was removed through centrifugation and buffer exchange. The labeled membranes and mitochondria were then sequentially extruded through 1000, 400, and 200 nm polycarbonate membranes and immediately examined using laser confocal microscopy.

For the analysis of membrane marker expression and co-localization after extrusion. AD-Mito-NPs were treated with CD90 antibody (Biolegend, 206105) and Mito-Tracker Red (Beyotime, C1035), and flow cytometry was performed to evaluate the colocalization of CD90 and Mito-Tracker Red.

Mitochondria and AD-Mito-NPs were stained with JC-1 and analyzed via flow cytometry following the manufacturer's instructions to evaluate changes in mitochondrial membrane potential before and after extrusion. To assess the stability of mitochondrial membrane potential under simulated *in vivo* temperature conditions, freshly prepared AD-Mito-NPs were incubated at 37 °C and collected at 1, 3, and 7 days post-preparation. Their polydispersity index (PDI), particle size, and zeta potential were measured using a Malvern Zetasizer Nano ZS90, and JC-1 staining combined with flow cytometry was performed to detect dynamic alterations in mitochondrial membrane potential. In addition, AD-Mito-NPs with or without Carbonyl cyanide 3-chlorophenylhydrazone (CCCP, MCE, HY-100941) pretreatment (10 μM for 30 min) were subjected to the Seahorse XF Cell Mito Stress Test Kit (Agilent Technologies, Seahorse Bioscience) according to the manufacturer's protocols to detect mitochondrial respiratory function. All triple replicates were derived from three independent preparations of AD-Mito-NPs.

### 2.6 Cell Counting Kit-8 (CCK-8) assay

AD-Mito-NPs were quantified using the BCA assay. Capsular fibroblasts were seeded at 5000 cells per well in 96-well plates and treated with 0, 0.1, 1, or 5 μg/mL of AD-Mito-NPs. After 24 h, cell viability was measured with the CCK-8 kit according to the manufacturer's instructions, and the concentration that resulted in the highest viability (1 μg/mL) was selected for further experiments.

### 2.7 Cellular uptake of AD-Mito-NPs

AD-Mito-NPs were double-labeled with Dil and Mito-Tracker Green to treat joint capsule fibroblasts. After 24 h of incubation, cellular fluorescence was observed via confocal microscopy.

Joint capsule fibroblasts were separately treated with Mito-Tracker Red-labeled free mitochondria and Mito-Tracker Red/CD90 double-labeled AD-Mito-NPs. Following 24 h of treatment, cellular fluorescence was observed via confocal microscopy.

### 2.8 Intracellular ATP level analysis

To assess intracellular ATP after exposure to AD-Mito-NPs, we seeded capsular fibroblasts at 20,000 cells per well in 24-well plates and treated them with IL-1β (10 ng/mL) and/or AD-Mito-NPs (1 μg/mL). Cells were collected, and ATP levels were measured using the ATP Assay Kit (Beyotime, S0026) following the manufacturer's instructions.

### 2.9 Bulk RNA sequencing and untargeted metabolomic analysis

After 24 h of treatment with IL-1β (10 ng/mL) and/or AD-Mito-NPs (1 μg/mL), capsular fibroblasts were trypsinized, washed twice with PBS, and snap-frozen in liquid nitrogen. The sample size per group was 3. Untargeted metabolomics was performed by Novogene using LC-MS. Pooled quality control samples were prepared by mixing equal volumes of all experimental specimens and injected before, during, and after sample acquisition to monitor instrument stability. Pearson correlation analysis and PCA of QC samples confirmed reliable data reproducibility. Raw LC-MS data were converted to mzXML format via ProteoWizard [33], and peak extraction, alignment and retention time correction were performed using XCMS [34]. Differential metabolites were screened with combined criteria: Student's t-test P-value < 0.05, variable importance in projection (VIP) > 1.0 from PLS-DA model, and fold change (FC) > 1.2 or < 0.833. Multivariate statistical analysis was conducted on the curated data through the NovoMagic platform.

Capsular fibroblasts were treated with IL-1β (10 ng/mL) and/or AD-Mito-NPs (1 μg/mL) for 24 h. Each group had three samples. Total RNA was then isolated and prepared for sequencing according to the manufacturer's protocol. Library construction and Illumina sequencing were performed by Novogene, and the raw reads were analyzed on the NovoMagic platform. The *Rattus norvegicus* reference genome version r_4.3.1 from Ensembl was used for read alignment. Clean sequencing reads were mapped to the rat reference genome utilizing HISAT2 software. The raw read counts per gene were quantified with FeatureCounts embedded in the Subread package. Gene expression normalization was conducted via the built-in normalization approach of DESeq, which corrects for sequencing depth and gene length effects. Differential expression analysis was carried out with DESeq2 based on a negative binomial generalized linear model. Genes with p-value ≤ 0.05 were identified as differentially expressed genes. The raw datasets have been deposited in the BioProject database (BioProject ID PRJNA1504511).

### 2.10 Post-uptake TEM analysis

After 24 h of treatment with IL-1β (10 ng/mL) and/or AD-Mito-NPs (1 μg/mL), capsular fibroblasts were scraped and fixed in 2.5% glutaraldehyde at 4 °C for 2 h. Following dehydration in graded ethanol, the samples were sectioned with an ultramicrotome and examined by TEM.

### 2.11 Metabolic substrate uptake analysis

Capsular fibroblasts were treated with IL-1β (10 ng/mL) and/or AD-Mito-NPs (1 μg/mL) in the presence of either the fluorescent fatty-acid analog BODIPY 558/568 C12 (Red-C12; Thermo Fisher Scientific; 1 μM) or the fluorescent glucose analog 2-NBDG (Thermo Fisher Scientific; 300 μM) for 24 h as previously described [35]. After collection, the cells were subjected to flow cytometric analysis. Data were processed and analyzed with FlowJo (version 10).

### 2.12 Capsular fibroblasts mitochondrial respiration analysis

Capsular fibroblasts pretreated with IL-1β (10 ng/mL) and/or AD-Mito-NPs were seeded at 20,000 cells per well in an XF24 microplate with 200 μL of medium. After 24 h, mitochondrial respiration was measured using the Seahorse XF Cell Mito Stress Test Kit (Agilent Technologies, Seahorse Bioscience) according to the manufacturer's instructions.

### 2.13 Intracellular and mitochondrial ROS detection

Capsular fibroblasts were treated with IL-1β (10 ng/mL), ADSC membrane, free mitochondria, and/or AD-Mito-NPs (1 μg/mL) for 24 h, then loaded with DCFH-DA for intracellular ROS and MitoSOX Red (Life Technologies) for mitochondrial ROS according to the manufacturer's instructions. Images were obtained using confocal microscopy, and fluorescence intensity was measured with ImageJ.

### 2.14 Live/dead staining

Capsular fibroblasts were treated with AD-Mito-NPs and IL-1β (10 ng/mL) for 1, 2, and 3 days. Live/Dead staining was then performed to evaluate the effect of AD-Mito-NPs on cell viability.

### 2.15 Immunofluorescence staining

Briefly, joint capsule fibroblasts exposed to AD-Mito-NPs and IL-1β (10 ng/mL) for 24 h were fixed with 4% paraformaldehyde, washed three times with PBS, and blocked for 1 h at room temperature in PBST containing 1% BSA and 0.1% Triton X-100. Primary antibodies against Collagen type I (Col1, Cat. No.: A24112), Collagen type III (Col3, Cat. No.: A3795), matrix metalloproteinase 9 (MMP9, Cat. No.: A25299), matrix metalloproteinase 13 (MMP13, Cat. No.: A11755), IL-6 (Cat. No.: A0286), IL-10 (Cat. No.: A2171) and tumor necrosis factor-alpha (TNF-α, Cat. No.: A21265) (all Abclonal, 1:300 dilution) were applied at 4 °C overnight. After PBS washes, species-matched Alexa Fluor-conjugated secondary antibodies (1:200) were incubated for 1 h at room temperature. F-actin (Actin-Tracker Green-488; Beyotime, C2201S) was visualized with phalloidin conjugate for 30 min, and nuclei were counterstained with DAPI. Images were captured on a laser scanning confocal microscope.

### 2.16 Cell migration assay

Primary capsular fibroblasts were seeded in 6-well plates. When they reached 90% confluence, the medium was replaced with 1% FBS, and the cells were exposed to IL-1β (10 ng/mL) and/or AD-Mito-NPs (1 μg/mL). A linear scratch was made using a 200 μL pipette tip. Phase-contrast images of the same fields were taken at 0 and 24 h with an inverted microscope. Migration rate = [(initial wound area − remaining area)/initial area] × 100%.

### 2.17 Animal model

All animal procedures were approved by the Animal Ethics Committee of the department. Age-matched rats were randomly assigned to experimental groups.

For small animal *in vivo* imaging, AD-Mito-NPs were labeled with DiR. The prepared AD-Mito-NPs (200 μg/mL) were injected into the joint cavity, and fluorescence imaging was performed at 1 h, 1 day, 3 days, 7 days, 10 days, and 14 days after injection using a small animal *in vivo* imaging system (In-Vivo Xtreme, BRUKER). The sample size consisted of 5 biological replicates.

The frozen-shoulder model was created by immobilizing the scapula to the humerus with a 1-0 nylon suture, as previously described [36,37]. Six weeks after surgery, 50 μL of either saline (control), AD-Mito-NPs (50 μL, 200 μg/mL), or Dexamethasone (Dex, 50 μL, 20 mg/mL Dexamethasone-Water Soluble, Sigma–Aldrich, D2915) was injected into the ipsilateral glenohumeral joint using an insulin syringe. The sample size consisted of 6 biological replicates. The injection dose of AD-Mito-NPs (10ug/injection) was determined based on preliminary experiments and relevant literature, with the dosage ranging from 10 to 20 μg [38,39].

The dexamethasone dose conversion was performed according to the following formula: Animal-Equivalent Dose (mg/kg) = Human Dose (mg/kg) × Km Ratio. The clinically effective intra-articular injection dose of dexamethasone ranges from 0.8 to 4 mg per patient, with a standard human body weight of 60 kg adopted for dose calculation. Based on a Km ratio of 5.3 for Sprague–Dawley rats with an average body weight of 250 g, the calculated rat-equivalent dose was 0.018–0.089 mg. In this study, water-soluble dexamethasone powder (containing approximately 65 mg pure dexamethasone per gram of powder) was used. The final injected dose of 0.065 mg per rat (0.26 mg/kg) fell within the converted clinical equivalent dose range.

Animals were euthanized eight weeks post-operatively for subsequent analyses.

### 2.18 Shoulder range of motion measurement

Shoulder range of motion was measured as previously described [37]. All range of motion measurements were performed by a single blinded experimenter who was unaware of the group assignments. Briefly, after isolating the shoulder girdle from the trunk, a constant torque of 3.92 × 10^−3^ N m (10 g weight) was applied to the distal humerus. The abduction angle was defined as the angle between the line of the scapular spine and the axis connecting the humeral head and the medial/lateral epicondyles, and it was recorded with a goniometer.

### 2.19 MRI analysis

Magnetic resonance imaging was conducted on a 7.0 T Bruker Biospec system (Germany). T2-weighted spin-echo sequences with fat suppression were acquired for each shoulder.

### 2.20 Histological analysis

The collected human joint capsule tissues were fixed in 4% paraformaldehyde for 24 h prior to dehydration treatment. The shoulder joints of rats were fixed in 4% paraformaldehyde, then decalcified in 0.5 M EDTA (pH 7.2–7.4) for 8 weeks. Tissues were dehydrated through a graded ethanol series, cleared in xylene, and embedded in paraffin blocks. Serial sections were cut at a thickness of 5 μm using a microtome, dewaxed, and rehydrated before staining. Histological staining was performed with hematoxylin and eosin (H&E) and Masson's trichrome following standard manufacturer protocols. Imaging was done under a brightfield light microscope equipped with a digital camera.

To assess potential systemic toxicity, major organs such as the heart, liver, spleen, lung, and kidney were processed similarly through fixation, paraffin embedding, and sectioning at 5 μm. These sections were stained with H&E and examined for structural integrity and abnormal changes under light microscopy.

### 2.21 Immunohistochemistry

Paraffin sections of the joint capsule were dewaxed, rehydrated, and treated with 3% H_2_O_2_ to quench endogenous peroxidase activity. After antigen retrieval, sections were permeabilized with 0.3% Triton X-100, blocked with 5% normal goat serum, and incubated overnight at 4 °C with primary antibodies including Col I (Cat. No.: A24112), Col III (Cat. No.: A3795), MMP9(Cat. No.: A25299), MMP13 (Cat. No.: A11755), IL-6 (Cat. No.: A0286), Cox4 (Cat. No.: A28231), Opa1 (Cat. No.: A9833), IL1 (Cat. No.: A27676) and TNF-α (Cat. No.: A21265) (all Abclonal, 1:300 dilution) which diluted in blocking buffer. Following PBS washes, HRP-conjugated secondary antibodies were applied for 1 h at 25 °C. Signals were developed with DAB, nuclei counterstained with hematoxylin, and sections mounted with neutral balsam. Images were captured under a light microscope.

### 2.22 Serum biochemistry

Blood was collected, centrifuged, and the serum was immediately analyzed on an automatic biochemical analyzer (BIOBASE BK280) for ALT, AST, TBIL, DBIL, creatinine, and urea.

### 2.23 Statistical analysis

Fluorescence intensity and capsule thickness were measured using Image J. All statistical analyses were conducted with GraphPad Prism 10.0. Data are shown as mean ± SD. Comparisons among three or more groups were analyzed with one-way ANOVA followed by Tukey's post-hoc test, while two-group comparisons were performed using a two-tailed two sample t test. P < 0.05 was considered statistically significant: *P < 0.05, **P < 0.01, ***P < 0.001, ****P<0.0001. Sample sizes (n) are indicated in each figure legend.

## 3 Results

### 3.1 Mitochondrial energy metabolism is impaired in patients with adhesive capsulitis

**Fig. 1:**
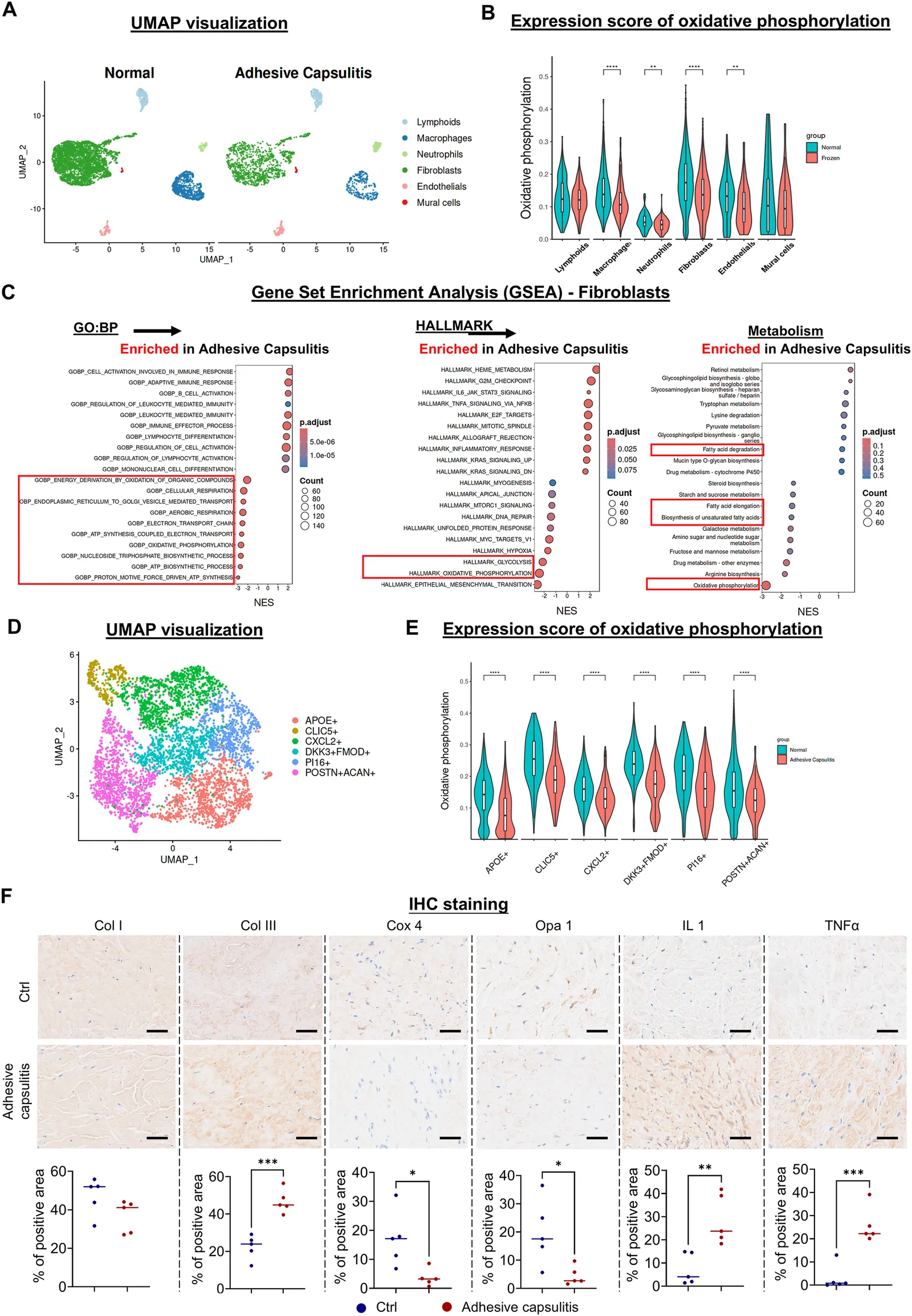
Single-cell transcriptomic analysis and immunohistochemical analysis of joint capsule tissues from normal individuals and patients with adhesive capsulitis. (A) UMAP visualization of all cells. (B) Oxidative phosphorylation scores of subpopulations. (C) GSEA of differentially expressed genes in fibroblasts. (D) UMAP visualization of fibroblasts. (E) Oxidative phosphorylation scores of fibroblast subpopulations. (F) Representative images of IHC staining and the semi-quantitative statistical analysis for Col I, Col III, Cox4, Opa1, IL1 and TNF a. Statistical significance was determined by two-tailed two sample t test. *P < 0.05, **P < 0.01, ***P < 0.001, ****P < 0.0001; n = 4–5.

To examine cell metabolic gene expression profiles in joint capsule tissues from clinical samples, we analyzed a public cohort containing single-cell RNA sequencing data of shoulder capsule tissues from patients with or without adhesive capsulitis [29]. The joint capsule cells were divided into 6 subgroups based on characteristic genes, including lymphocytes, macrophages, neutrophils, fibroblasts, endothelial cells, and mural cells (Fig. 1A and S1A). Fibroblasts were the most common cell type. For most cell types (macrophages, neutrophils, fibroblasts, and endothelial cells), the expression score of oxidative phosphorylation was significantly decreased in patients with adhesive capsulitis (Fig. 1B and S1B). Differential gene analysis and Gene Set Enrichment Analysis (GSEA) revealed that pathways such as the electron transport chain, ATP synthesis, oxidative phosphorylation, and fatty acid metabolism were significantly enriched, in fibroblasts of adhesive capsulitis tissues relative to comparator patient tissues, indicating improvement of energy metabolism (Fig. 1C and S2A). In fibroblast subpopulations, genes related to ATP synthesis and oxidative phosphorylation were all significantly downregulated in adhesive capsulitis specimens, accompanied by a significant reduction in oxidative phosphorylation scores (Fig. 1D, E and S2B-D). In human adhesive capsulitis tissues, the expression of type III collagen was significantly upregulated, suggesting the formation of abnormal collagen structures (Fig. 1F). Furthermore, the expression levels of pro-inflammatory cytokines IL-1 and TNF-α were increased (Fig. 1F). In addition, the oxidative phosphorylation regulatory protein COX4 and the inner mitochondrial membrane protective protein OPA1 were significantly downregulated (Fig. 1F). These findings suggest substantial dysfunction in mitochondrial energy metabolism in the joint capsule fibroblasts of affected patients.

### 3.2 Characteristic of AD-Mito-NPs

**Fig. 2:**
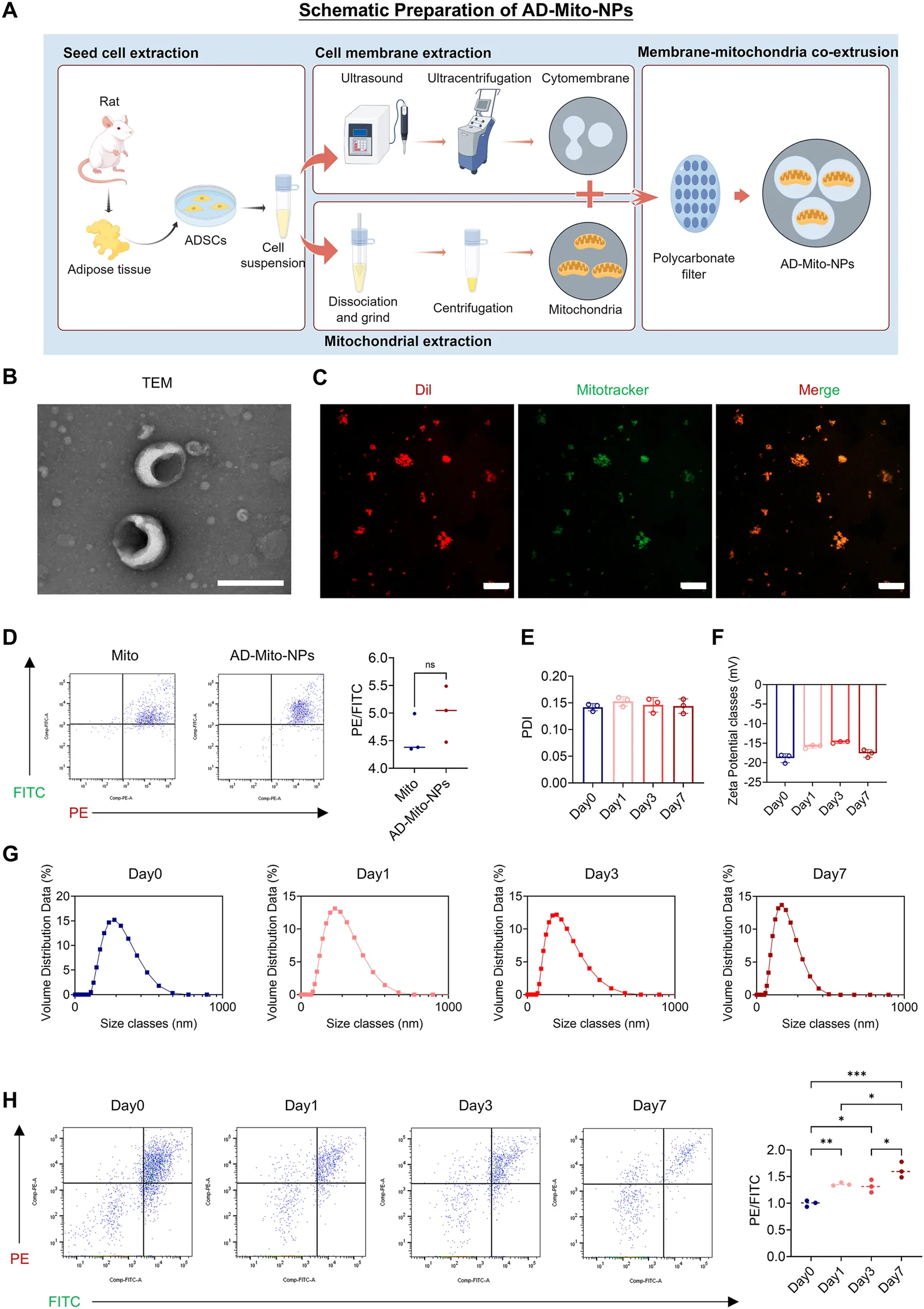
Characterization and *in vitro* stability evaluation of AD-Mito-NPs. (A) Schematic illustration of the extraction and culture of ADSCs, isolation of cell membranes and mitochondria, and preparation of AD-Mito-NPs (created by figdraw.com; Copyright Code: YUUPW4df5d). (B) Representative TEM images of AD-Mito-NPs. Scale bar, 200 nm. (C) Representative confocal images of AD-Mito-NPs. The ADSC membrane was pre-labeled with Dil (red), and mitochondria were pre-labeled with MitoTracker (green) before extrusion. Scale bar, 20 μm. (D) Flow cytometric quantification of mitochondrial membrane potential in mitochondria (Mito) and AD-Mito-NPs via JC-1 staining (n = 3). (E-G) PDI, zeta potential and size distribution of AD-Mito-NPs at different time points (0, 1, 3, and 7 days) after synthesis (E, F, n = 3). (H) Flow cytometric quantification of mitochondrial membrane potential in AD-Mito-NPs at different time points (0, 1, 3, and 7 days) after synthesis via JC-1 staining (n = 3). Data are presented as mean ± SD. Statistical significance was determined by two sample t test (D) or one-way ANOVA (H) with Tukey's multiple comparisons test. *P < 0.05, **P < 0.01, ***P < 0.001.

Cell membranes and mitochondria were isolated and extracted from cultured ADSCs. The AD-Mito-NPs were assembled using the membrane extrusion method (Fig. 2A). TEM images showed the vesicular structure of AD-Mito-NPs, displaying a typical spherical or saucer-shaped form (Fig. 2B). Confocal imaging showed obvious co-localization between Dil-labeled ADSC membranes and Mito-Tracker Green-labeled mitochondria (Fig. 2C). Flow cytometry revealed that approximately 75% of the particles were dual-positive for the mitochondrial probe Mito-Tracker and the stem cell membrane marker CD90 (Fig. S3). Compared with free mitochondria, AD-Mito-NPs exhibited no significant changes in mitochondrial membrane potential as revealed by JC1 staining (Fig. 2D). When incubated at 37 °C, AD-Mito-NPs exhibited stable particle size at 1, 3 and 7 days after preparation, accompanied by a low, steady PDI value (roughly 0.12–0.15) and homogeneous particle size distribution (predominantly 150–400 nm). The zeta potential fluctuated moderately over the 7-day *in vitro* incubation period, ranging from −15 mV to −20 mV (Fig. 2E–G). Intriguingly, the PE/FITC ratio from JC-1 staining (an indicator of mitochondrial membrane potential) did not decline progressively from day 1 to day 7; instead, this ratio increased slightly during the incubation (Fig. 2H).

### 3.3 Uptake patterns of AD-Mito-NPs

**Fig. 3:**
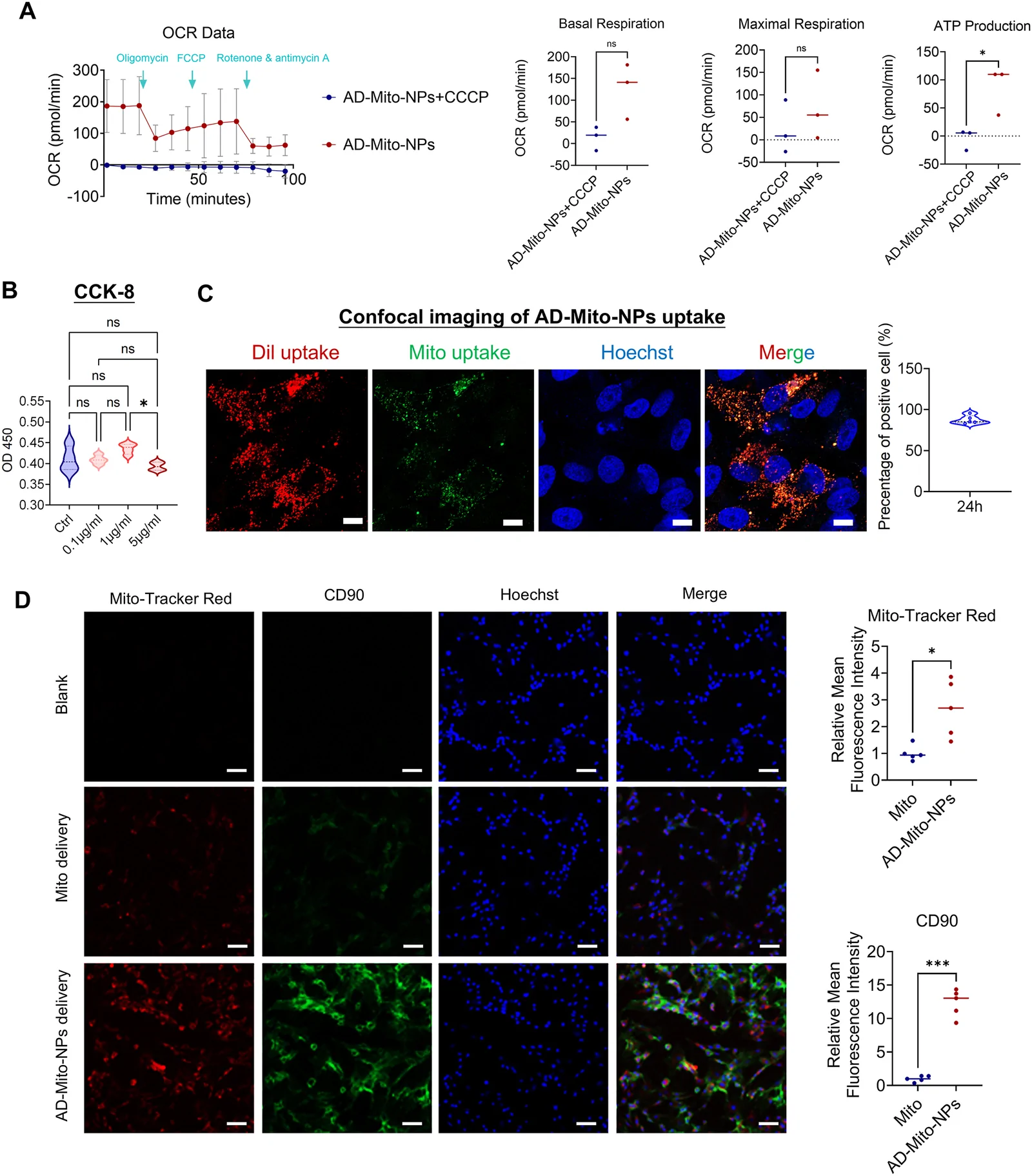
Oxygen consumption rate (OCR) and joint capsule fibroblast uptake of AD-Mito-NPs. (A) Seahorse XF analysis of OCR in AD-Mito-NPs and CCCP-pretreated AD-Mito-NPs, with statistical analysis of key mitochondrial metabolic parameters (n = 3). (B) Viability of joint capsule fibroblasts treated with different concentrations of AD-Mito-NPs for 24 h, assessed by CCK-8 assay (n = 4–5). (C) Representative confocal images and quantitative analysis of cellular uptake of Dil (red)- and MitoTracker (green)-labeled AD-Mito-NPs (1 μg/ml) in joint capsule fibroblasts (n = 5). Blue: Hoechst (nucleus). Scale bar, 20 μm. (D) Representative confocal images and quantitative analysis of cellular uptake of CD90 (green)- and MitoTracker (red)-labeled AD-Mito-NPs (1 μg/ml) or MitoTracker (red)-labeled mitochondria (Mito) in joint capsule fibroblasts (n = 5). Blue: Hoechst (nucleus). Scale bar, 50 μm. Data are presented as mean ± SD. Statistical significance was determined by two sample t test (A and D) or one-way ANOVA (B) with Tukey's multiple comparisons test.

Seahorse oxidative phosphorylation analysis confirmed that AD-Mito-NPs exhibited normal respiratory function (Fig. 3A). Treatment with the mitochondrial uncoupler CCCP abolished the respiratory capacity of AD-Mito-NPs (Fig. 3A). The *in vitro* CCK-8 assay demonstrated that 1 μg/mL was the safe concentration of AD-Mito-NPs for joint capsule fibroblasts (Fig. 3B). A concentration of 5 μg/mL may consume substantial energy substrates, thereby reducing cell viability instead. After 24 h of incubation, the cellular uptake efficiency of AD-Mito-NPs reached approximately 85% (Fig. 3C). Compared with the direct delivery of free mitochondria, CD90-FITC antibody/Mito-Tracker Red dual-labeled AD-Mito-NPs showed significantly higher fluorescence intensity in fibroblasts, indicating significantly improved mitochondrial internalization (Fig. 3D). Collectively, these findings confirm that AD-Mito-NPs retain biological activity and effectively enhance the cellular uptake of mitochondria by fibroblasts.

### 3.4 AD-mito-NPs treatment alters the gene transcription and metabolic phenotype of fibroblasts

**Fig. 4:**
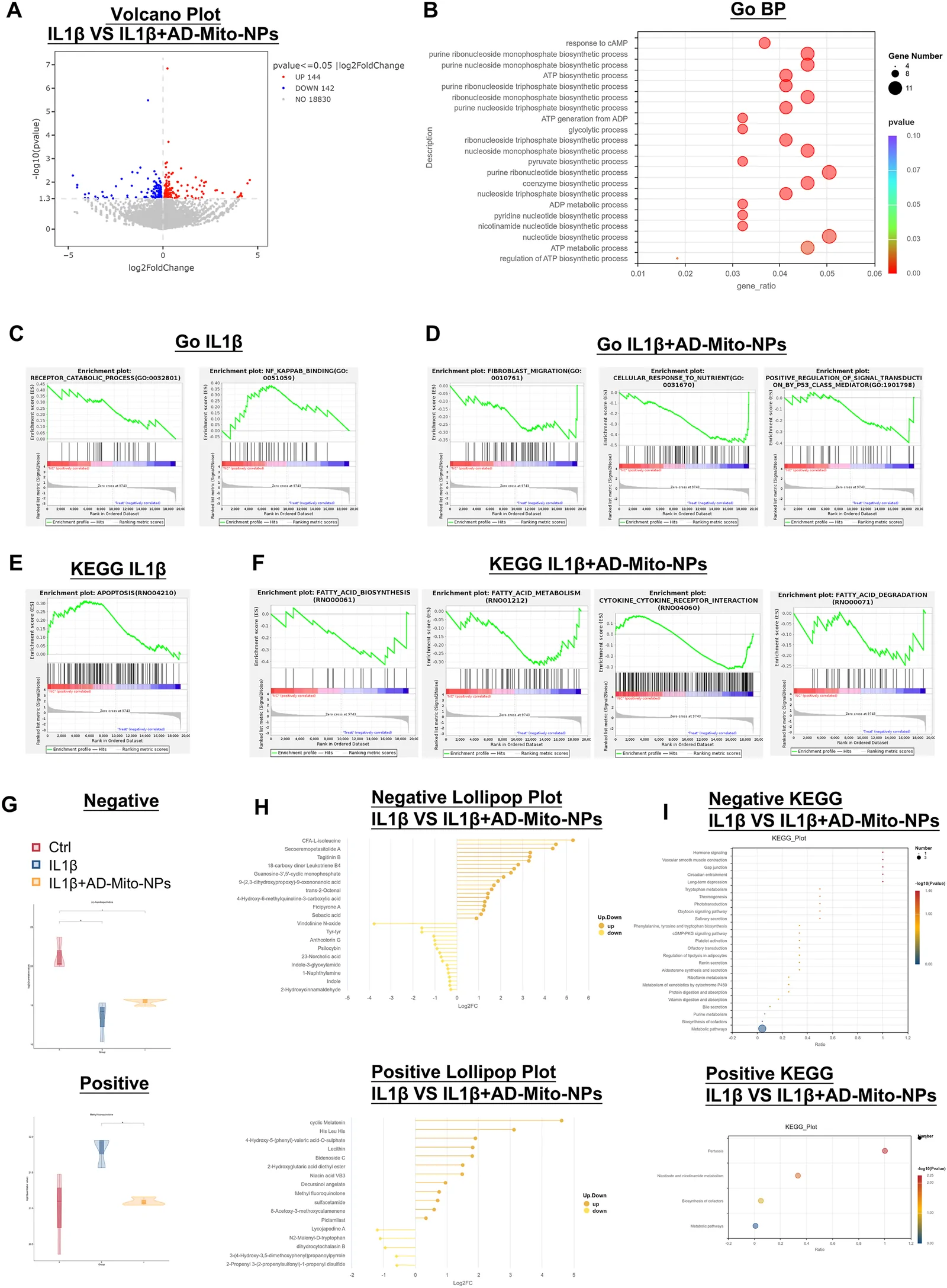
Transcriptomic and metabolomic profiling of joint capsule fibroblasts treated with AD-Mito-NPs. (A) Volcano plots depicting differentially expressed genes between IL-1β (10 ng/mL) treatment and IL-1β plus AD-Mito-NPs co-treatment. (B) Bubble diagram of enriched biological processes between IL-1β (10 ng/mL) treatment and IL-1β plus AD-Mito-NPs co-treatment. (C) Gene Set Enrichment Analysis (GSEA) of Gene Ontology (GO) terms for signaling pathways upregulated in the IL-1β treatment group. (D) GSEA of GO terms for signaling pathways upregulated in the IL-1β plus AD-Mito-NPs co-treatment group. (E) GSEA of KEGG pathways upregulated in the IL-1β treatment group. (F) GSEA of KEGG pathways upregulated in the IL-1β plus AD-Mito-NPs co-treatment group. (G) Violin plots showing differential metabolites under negative and positive ion modes. (H) Lollipop plot illustrating differential metabolites in negative and positive ion modes. (I) KEGG pathway enrichment analysis of differential metabolites in negative and positive ion modes.

Joint capsule fibroblasts were treated with IL-1β to investigate their metabolic abnormalities in an inflammatory state. RNA-Seq analysis showed that a total of 286 genes changed significantly (144 upregulated and 142 downregulated) after treatment with AD-Mito-NPs (Fig. 4A). These differentially expressed genes were mainly enriched in energy metabolism pathways, indicating that delivering AD-Mito-NPs caused improvement of energy metabolism (Fig. 4B). GSEA analysis of GO pathways revealed that cellular catabolic processes and the NF-kappa B (NF-κB) binding signaling pathway, which are related to inflammation, were significantly enriched in the IL-1β treatment group (Fig. 4C). Conversely, treatment with AD-Mito-NPs significantly promoted the migration ability, cellular response to nutrients, and signal transduction processes (Fig. 4D). KEGG analysis further showed that IL-1β exposure increased the apoptosis pathway. Treatment with AD-Mito-NPs significantly boosted mitochondrial metabolism—especially fatty acid metabolism—and cytokine-receptor interaction pathways (Fig. 4E and F). Untargeted metabolomics revealed the dynamic changes in metabolites caused by IL-1β and the regulatory effects of AD-Mito-NPs. In negative ion mode, metabolites altered by IL-1β were mainly decreased; in positive ion mode, they were mostly increased (Fig. 4G). Treatment with AD-Mito-NPs tended to reverse inflammation-related metabolic changes in both modes (Fig. 4H and I). These regulated metabolites were primarily involved in hormone synthesis, cGMP-PKG signaling, protein digestion and absorption, metabolic pathways, and cofactor biosynthesis (Fig. 4H and I). These findings suggest that AD-Mito-NPs partially rescued metabolic impairment fibroblast energy metabolism, which in turn affects key cellular processes, including cell migration, inflammatory responses, and signal transduction.

### 3.5 AD-mito-NPs enhances mitochondrial energy metabolism in fibroblasts

**Fig. 5:**
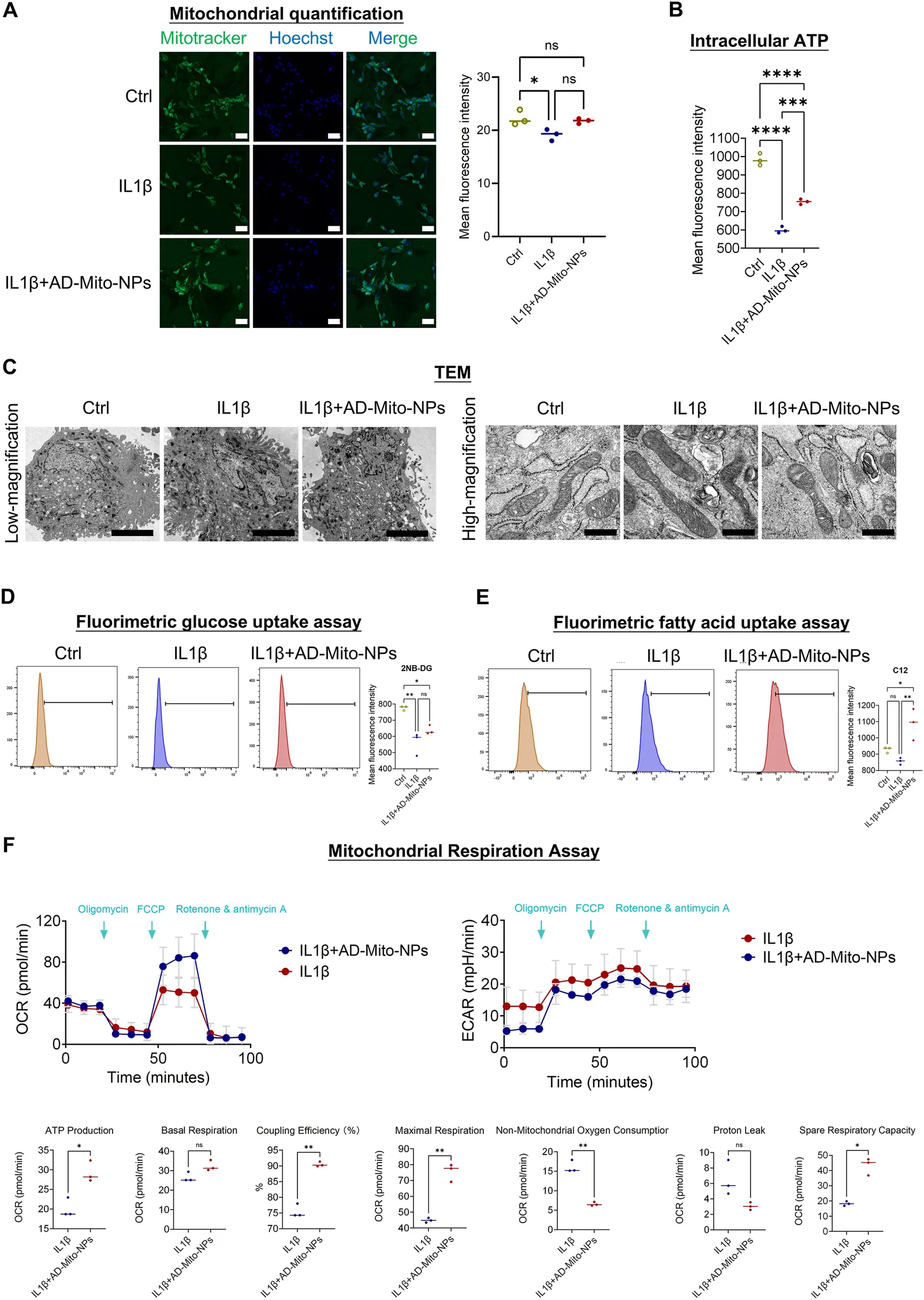
Analysis of mitochondrial metabolism in joint capsule fibroblasts treated with AD-Mito-NPs. (A) Representative confocal images and quantification of mean fluorescence intensity of MitoTracker Green staining in fibroblasts treated with IL-1β (10 ng/mL) and/or AD-Mito-NPs. Green: MitoTracker (mitochondria); Blue: Hoechst (nuclei). Scale bar, 50 μm; n = 3. (B) Quantification of cellular ATP content across treatment groups, measured by bioluminescence assay; n = 3. (C) TEM images depicting mitochondrial ultrastructure. Scale bar, 5 μm (Low-magnification); 500 nm (High-magnification). (D) Flow cytometry analysis and quantification of cellular 2-NBDG uptake; n = 3. (E) Flow cytometry analysis and quantification of C12 fatty acid uptake; n = 3. (F) Seahorse XF analysis of OCR and extracellular acidification rate (ECAR) profiles, with statistical analysis of key mitochondrial metabolic parameters; n = 3. Data are presented as mean ± SD. Statistical significance was determined by two sample t test (F)or one-way ANOVA with Tukey's multiple comparisons test (A, B, D, E). *P < 0.05, **P < 0.01, ***P < 0.001, ****P< 0.0001.

We further investigated the effects of AD-Mito-NPs on mitochondrial energy metabolism in fibroblasts. Using MitoTracker fluorescence labeling and confocal microscopy, we found that IL-1β treatment significantly decreased fluorescence intensity. Conversely, treatment with AD-Mito-NPs partially restored it to levels similar to those in untreated controls (Fig. 5A). Intracellular ATP assays revealed that IL-1β significantly reduced ATP production, whereas AD-Mito-NPs treatment effectively increased ATP levels (Fig. 5B). TEM revealed that IL-1β exposure caused severe ultrastructural damage to mitochondria, including disrupted cristae, fragmentation, and vacuolar degeneration, indicating serious impairment of the inner membrane system (Fig. 5C). In contrast, AD-Mito-NPs intervention partly restored cristae structure and reduced these abnormal changes (Fig. 5C). Flow cytometry analysis demonstrated that IL-1β treatment impaired the cellular uptake of a fluorescent glucose analogue (2-NBDG) and a fatty acid probe (C12-BODIPY) (Fig. 5D and E). Supplementing with AD-Mito-NPs significantly increased the uptake of both substrates, especially the fatty acid probe. Moreover, seahorse extracellular flux (XF) analysis revealed that AD-Mito-NPs treatment significantly enhanced mitochondrial respiration, increasing both maximum respiratory capacity and ATP-linked respiration (Fig. 5F). These findings confirm that oxidative phosphorylation in fibroblasts is enhanced by AD-Mito-NPs.

#### 3.5.1 AD-mito-NPs promotes fibroblast survival and reduced inflammation

**Fig. 6:**
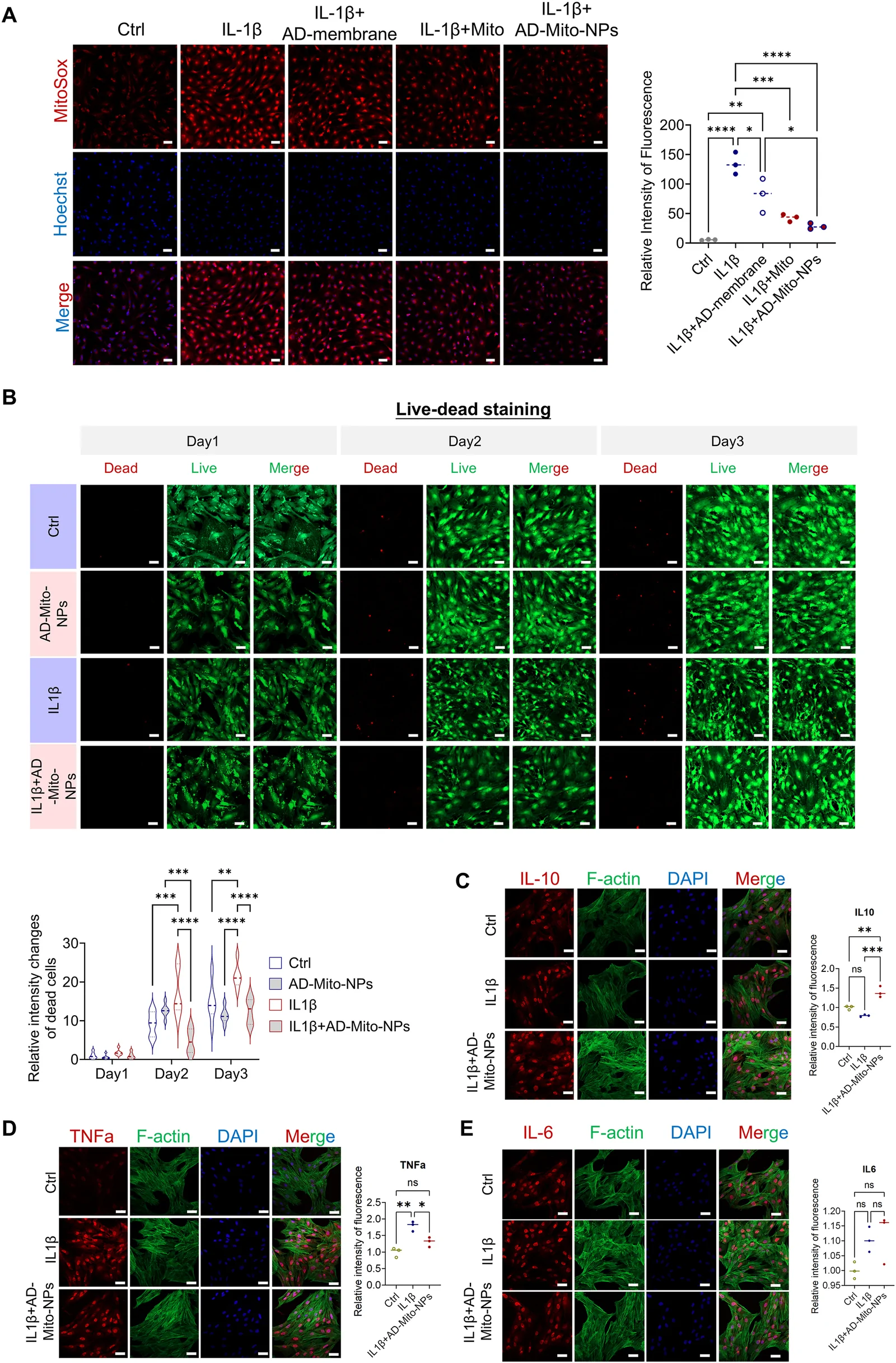
Analysis of oxidative stress, cell viability, and inflammation levels in joint capsule fibroblasts treated with AD-Mito-NPs. (A) Representative confocal images and quantification of mitochondrial superoxide levels detected by MitoSOX Red fluorescence. Red: MitoSOX (mitochondrial superoxide); Blue: Hoechst (nuclei). Scale bar, 50 μm. n = 3. (B) Representative confocal images and quantification of cell viability assessed by live/dead staining. Green: Calcein-AM (live cells); Red: PI (dead cells). Scale bar, 50 μm. n = 6. (C-E) Representative immunofluorescence images and quantification of fluorescence intensity for IL-10 (C), TNF-α (D), and IL-6 (E) expression. Red: target cytokine; Green: F-actin (Phalloidin); Blue: DAPI (nuclei). Scale bar, 50 μm. n = 3. Data are presented as mean ± SD. Statistical significance was determined by one-way ANOVA (A, C, D and E) or two-way ANOVA (B) with Tukey's multiple comparisons test. *P < 0.05, **P < 0.01, ***P < 0.001, ****P< 0.0001.

The effectiveness of AD-Mito-NPs in reducing inflammation-related mitochondrial dysfunction and oxidative stress was assessed by examining their impact on fibroblast oxidative damage, viability, and inflammation. Confocal imaging showed that IL-1β stimulation significantly increased levels of both intracellular total ROS, detected by DCFH-DA, and mitochondrial superoxide, labeled by MitoSOX (Fig. 6A and S4). Treatment with AD-Mito-NPs effectively decreased the production of these ROS species, and this effect was more significant compared with the direct delivery of ADSC membranes or free mitochondria (Fig. 6A). Because oxidative stress promotes apoptosis and damages cell survival, the observed reduction in ROS indicates a protective role for AD-Mito-NPs in preserving cell viability. Live/dead staining showed that IL-1β increased cell death, whereas AD-Mito-NPs reduced the proportion of dead cells on days 2 and 3 (Fig. 6B). Moreover, since IL-1β and the resulting oxidative stress can worsen inflammation, we evaluated key cytokines through immunofluorescence (Fig. 6C–E). AD-Mito-NPs treatment notably increased the expression of the anti-inflammatory cytokine IL-10 and decreased the production of the pro-inflammatory cytokine TNF-α.

### 3.6 AD-mito-NPs promotes fibroblast migration and regulated ECM function

**Fig. 7:**
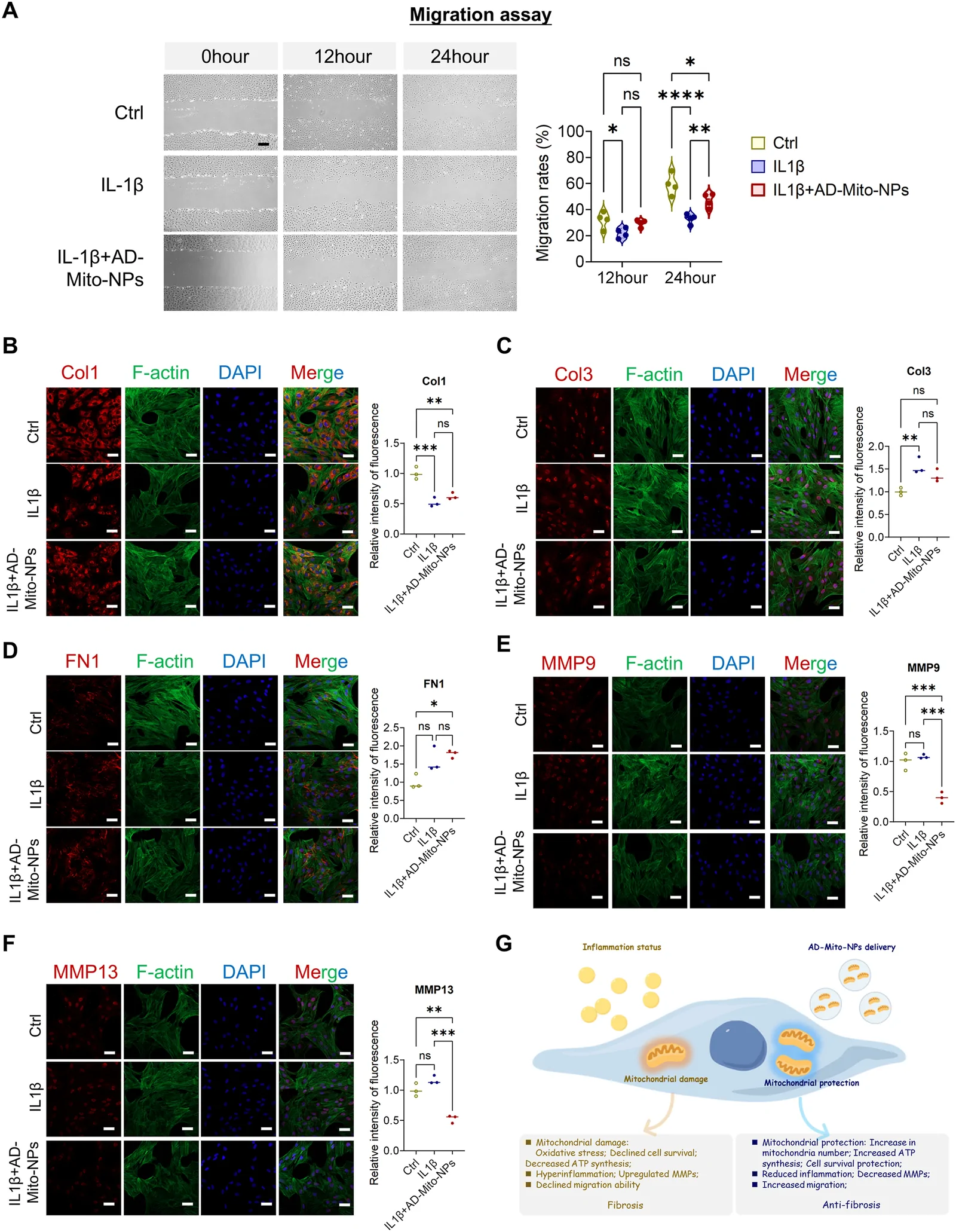
Effects of AD-Mito-NPs on migration and ECM-related protein expression in joint capsule fibroblasts. (A) Representative images and quantification of cell migration assessed by wound healing assay. Scale bar, 100 μm. n = 4. (B–F) Representative immunofluorescence images (left) and quantification of fluorescence intensity (right) for collagen I (Col1, B), collagen III (Col3, C), fibronectin 1 (FN1, D), matrix metalloproteinase 9 (MMP9, E), and matrix metalloproteinase 13 (MMP13, F). Red: target protein; Green: Phalloidin (F-actin); Blue: DAPI (nuclei). n = 3. Scale bar, 50 μm. (G) Schematic diagram illustrating phenotypic changes in fibroblasts following inflammatory stimulation and AD-Mito-NPs delivery. Data are presented as mean ± SD. Statistical significance was determined by one-way ANOVA (B–F) or two-way ANOVA (A) with Tukey's multiple comparisons test. *P < 0.05, **P < 0.01, ***P < 0.001, ****P<0.0001.

Energy metabolism acts as a vital adaptive mechanism influencing cellular migration and ECM remodeling [40]. Treatment with AD-Mito-NPs partially reversed IL-1β-induced inhibition of fibroblast migration, as shown by the cell scratch assay (Fig. 7A). Immunofluorescence analysis further revealed that IL-1β stimulation suppressed type I collagen production, while increasing the expression of type III collagen and fibronectin 1 (FN1) (Fig. 7B–D). In contrast, although no statistically significant difference was observed, AD-Mito-NPs treatment elevated the mean fluorescence intensity of type I collagen while lowering the mean fluorescence intensity of type III collagen. Additionally, AD-Mito-NPs treatment markedly decreased the synthesis of MMP13 and MMP9 (Fig. 7E and F). These results suggest that AD-Mito-NPs reduce abnormal fibrosis *in vitro*.

### 3.7 AD-mito-NPs transplantation improves shoulder joint function in rats with adhesive capsulitis

**Fig. 8:**
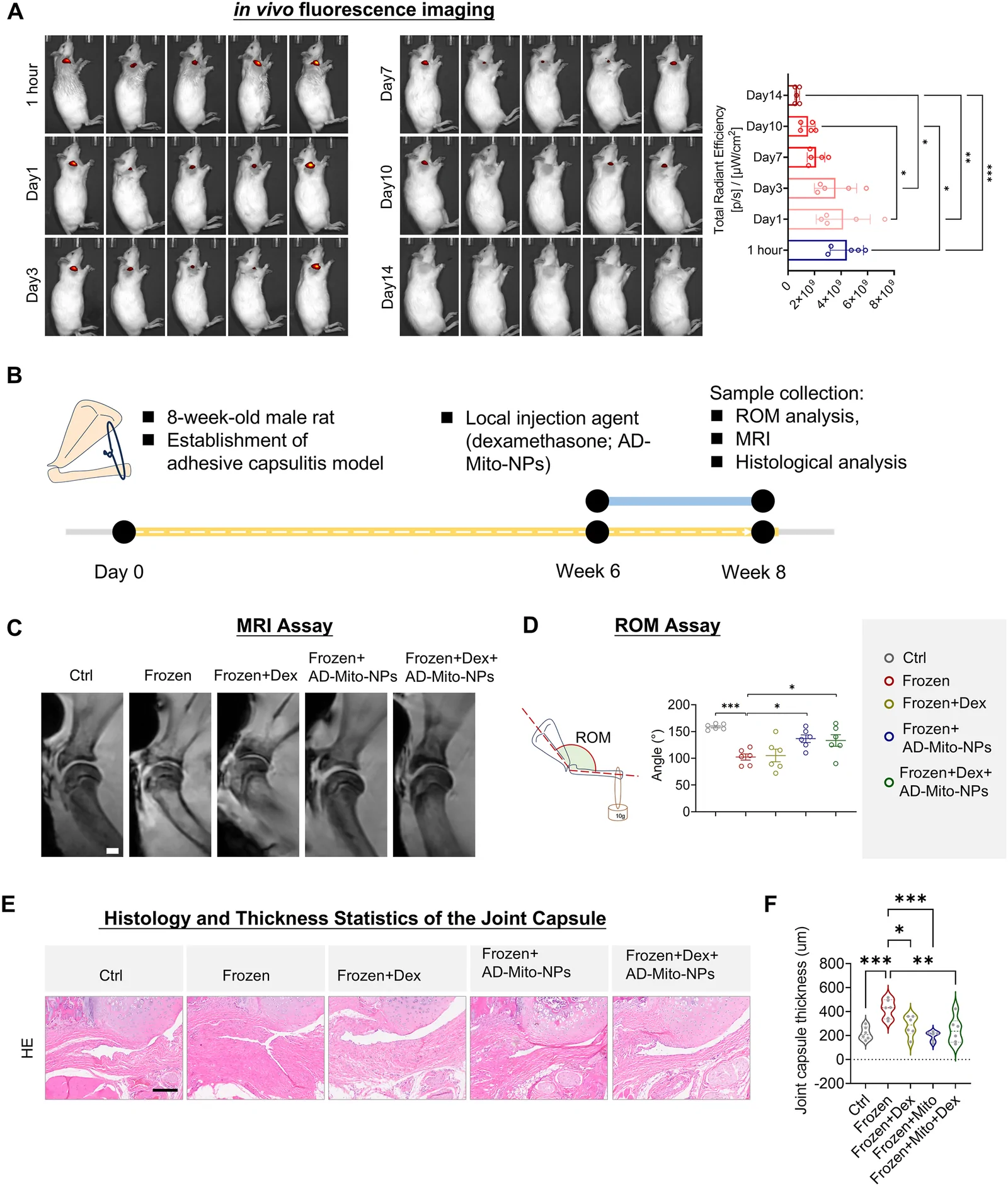
Effects of AD-Mito-NPs and Dex on the progression of adhesive capsulitis in rats. (A) *In vivo* fluorescence imaging and quantitative analysis of total radiant efficiency of DiR-labeled AD-Mito-NPs at different days after intra-articular shoulder injection (n = 5). (B) Schematic diagram illustrating the experimental timeline of adhesive capsulitis model induction and the subsequent intra-articular administration of AD-Mito-NPs, Dex or vehicle control. (C) Representative MRI of the shoulder joint. Scale bar, 2 mm. (D) Statistical analysis of glenohumeral joint range of motion (ROM); n = 6. (E-F) Representative images of HE staining (E) with quantitative analysis (F) of capsular thickness. n = 6. Scale bar, 200 μm. Data are presented as mean ± SD. Statistical significance was determined by two-way ANOVA (A) or one-way ANOVA (D and F) with Tukey's multiple comparisons test. *P < 0.05, **P < 0.01, ***P < 0.001, ****P < 0.0001.

Small animal *in vivo* imaging was performed to investigate the intra-articular retention of AD-Mito-NPs. The results showed that fluorescence signal was still detectable in the joint cavity 10 days after a single injection, while disappeared in day 14 (Fig. 8A). To assess the therapeutic potential of AD-Mito-NPs for adhesive capsulitis, a rat model was created, and intra-articular injections of AD-Mito-NPs and/or Dex were administered at the 6th week after shoulder immobilization (Fig. 8B). Magnetic resonance imaging (MRI) showed patchy high-signal areas in the shoulder joints of the adhesive capsulitis group, indicating inflammatory infiltration and tissue edema. After treatment with AD-Mito-NPs, both the size and brightness of these hyperintense signals were significantly reduced (Fig. 8C). After 8 weeks of immobilization, the range of motion in the model group decreased significantly to approximately 105°, compared to around 160° in healthy shoulders. Dex treatment did not improve shoulder range of motion at the 8th week after shoulder immobilization, while AD-Mito-NPs injection caused a significantly enhancement (Fig. 8D). Additionally, combining Dex and AD-Mito-NPs did not show better results than AD-Mito-NPs alone. H&E staining revealed considerable thickening of the articular capsule in the adhesive capsulitis (frozen) group (Fig. 8E and F). Both Dex and AD-Mito-NPs treatment significantly reduced capsular thickening (Fig. 8E and F).

**Fig. 9:**
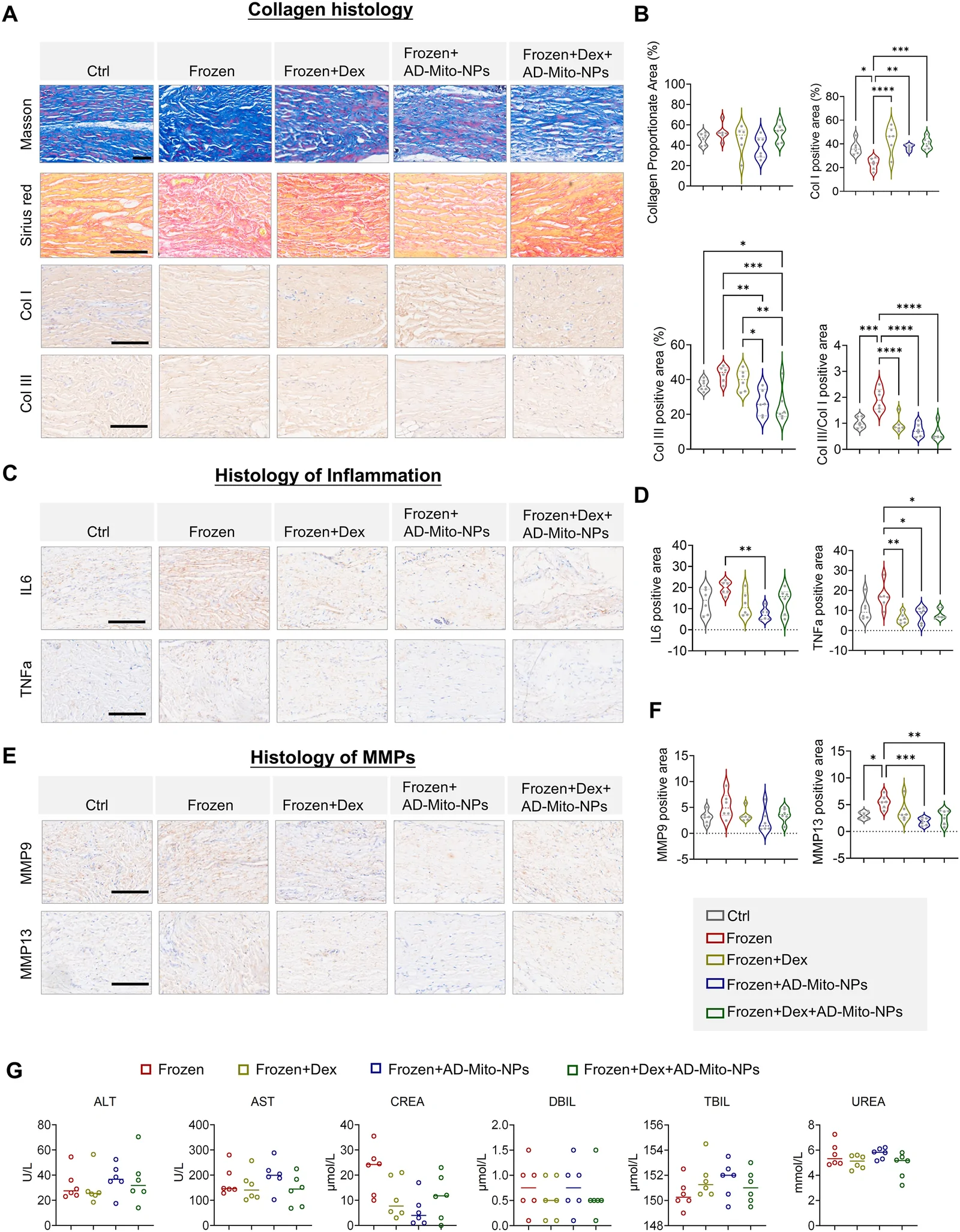
IHC analysis of adhesive capsulitis tissues in rats treated with AD-Mito-NPs or Dex and serum biochemical indicators analysis. (A-B) Histological evaluation of collagen deposition and organization in the joint capsule. (A) Representative images of Masson's trichrome staining, picrosirius red staining, and IHC staining for Col I and Col III. Scale bar, 100 μm. (B) Semi-quantitative statistical analysis of collagen content and the Col1/Col3 ratio based on IHC and histological stains. (C-D) Assessment of inflammatory infiltration. (C) Representative IHC images of pro-inflammatory cytokines IL-6 and TNF-α. Scale bar, 100 μm. (D) Semi-quantitative statistical analysis of positive area based on IHC staining. (E-F) Analysis of matrix metalloproteinases (MMPs) expression. (E) Representative IHC images of MMP9 and MMP13. Scale bar, 100 μm. (F) Semi-quantitative statistical analysis of positive area based on IHC staining. (G) Quantitative analysis of key serum biochemical indicators. n = 6. Data are presented as mean ± SD. Statistical significance was determined by one-way ANOVA (B, D, F and G) with Tukey's multiple comparisons test. *P < 0.05, **P < 0.01, ***P < 0.001, ****P < 0.0001.

Masson trichrome and Sirius Red staining showed increased collagen deposition with disorganized and haphazard fiber arrangement in the adhesive capsulitis group (Fig. 9A and B). Immunohistochemical staining also demonstrated higher expression of type III collagen along with a decrease in type I collagen, indicating a shift in collagen composition typical of fibrotic progression (Fig. 9A and B). Both the Dex injection group and the AD-Mito-NPs transplantation group exhibited a reversal of this trend, characterized by increased type I collagen expression and decreased type III collagen levels. Notably, the AD-Mito-NPs transplantation group had a lower ratio of type III to type I collagen and a more organized, continuous fiber network compared to other treatment groups.

Regarding inflammatory factors, elevated levels of TNF-α and IL-6 were observed in the adhesive capsulitis model group (Fig. 9C and D). Both cytokines decreased after treatment with Dex and AD-Mito-NPs. IHC staining also showed significantly higher expression of MMP-9 and MMP-13 in the adhesive capsulitis model group (Fig. 9E and F). AD-Mito-NPs demonstrated a stronger inhibitory effect, especially reducing MMP-13 expression, which aligns with the *in vitro* results.

Safety evaluations showed that administering AD-Mito-NPs did not cause any significant changes in serum biochemical parameters (Fig. 9G). No significant histological differences were observed in major organs between the AD-Mito-NPs injection group and the untreated control group (Fig. S5). Overall, AD-Mito-NPs treatment improves the histological structure of the joint capsule, restores collagen balance, reduces inflammatory responses, and corrects ECM metabolic imbalance in a rat model of adhesive capsulitis.

## 4 Discussion

In this study, we developed a therapeutic approach using AD-Mito-NPs. These nanoparticles showed high cellular uptake efficiency *in vitro*. Treatment with AD-Mito-NPs effectively reversed IL-1β-induced metabolic dysregulation in fibroblasts. In a rat model of adhesive capsulitis, local administration of AD-Mito-NPs significantly improved the range of motion of the shoulder joint and reduced the thickness of capsular fibrosis. These therapeutic effects are primarily due to enhanced mitochondrial bioenergy production, suppression of oxidative stress and apoptosis, downregulation of pro-inflammatory factors, and restoration of ECM remodeling balance. This combined action ultimately improves cellular function and normalizes collagen composition.

Current first-line treatments for adhesive capsulitis, including physical therapy, non-steroidal anti-inflammatory drugs (NSAIDs), and corticosteroid injections, mainly aim to relieve symptoms rather than fundamentally reverse the underlying pathology. Their effectiveness is often temporary and inconsistent, and some patients may experience ongoing pain and long-term functional impairment [4,[8], [9], [10]]. Developing treatments that halt and reverse the key disease mechanisms is an urgent clinical need to promote tissue repair and restore joint function. Based on single-cell transcriptomic profiling of clinical samples, we identified mitochondrial metabolic dysfunction as a central factor driving the inflammation-fibrosis cascade in adhesive capsulitis. Instead of merely suppressing symptoms, the developed AD-Mito-NPs target multiple pathological processes simultaneously, thereby normalizing ECM synthesis and breakdown, which reduces fibrosis and supports functional recovery.

Biomaterials designed for metabolic regulation precisely modulate the physiological states of diseased cells to control their fate and behavior, with mitochondria serving as the central orchestrators of cellular energy and metabolic homeostasis, and thus as critical therapeutic targets [41,42]. Although previous studies have shown that externally delivered mitochondria can enhance tissue repair, they often fail to integrate into the endogenous mitochondrial network of host cells and are instead eliminated through mitophagy following internalization [43]. Existing studies have shown that erythrocyte membrane-coated mitochondria exhibit desirable stability and are capable of fusing with endogenous mitochondria [44]. Herein, ADSC-derived membranes and isolated mitochondria were co-extruded to generate AD-Mito-NPs. The ADSC membrane may act as a native barrier to mitigate extracellular enzymatic breakdown and immune clearance of loaded mitochondria [45]. Its natural components significantly reduce foreign body reactions and prevent inflammatory side effects typically induced by synthetic materials. These ADSC membranes retain surface adhesion molecules that enable targeted enrichment at inflammation/fibrosis sites—where corresponding ligands are highly expressed [45,46]. Prepared using an extrusion method, the nanoparticles demonstrate a uniform size distribution. This property facilitates efficient tissue diffusion and enhances cellular uptake through endocytic pathways. Following delivery, the delivered mitochondria appear to participate in the energy metabolism of host cells. This integration enhances both oxidative phosphorylation and basal energy metabolism, an effect directly confirmed by Seahorse analysis and ATP measurements.

Reducing mitochondrial-derived ROS significantly alleviates damage to cellular DNA, proteins, and lipids caused by mitochondrial oxidative stress [15,[47], [48], [49]]. Normalized energy metabolism enables cells to maintain their normal immunomodulatory functions [16,50]. AD-Mito-NPs inhibit mitochondrial pathway apoptosis through metabolic regulation and improve fibroblast survival in inflammatory environments. AD-Mito-NP treatment promoted type I collagen synthesis (which provides strength [51,52]) while decreasing type III collagen (scar-related ([51,52]), and also downregulated matrix-degrading enzymes MMP9 and MMP13. This combined effect restored ECM homeostasis without causing pathological fibrosis.

Literature has demonstrated that the adhesive capsulitis model based on shoulder immobilization presents inflammation-dominated pathological changes within 1-2 weeks, corresponding to the early adhesive stage of frozen shoulder, while inflammation subsides at 3-6 weeks and shifts to a fibrosis-dominated frozen stage [36,37]. Accordingly, we performed *in vivo* intervention at 6 weeks of immobilization to investigate the therapeutic effects on tissue fibrosis. We verified in *in vitro* experiments that AD-Mito-NPs exhibited superior cellular uptake and regulatory effects on mitochondrial oxidative stress compared with the ADSC membrane and free mitochondria control groups. Whether AD-Mito-NPs possess better *in vivo* uptake capacity and mitochondrial modulatory function than the two control groups will be investigated in future studies. Injection of potent synthetic glucocorticoids is commonly used to manage inflammation and related pain, but there is no consensus regarding the optimal formulation, dosage, or treatment efficacy [53]. The dosage administered to rats in this study was converted from doses used in clinical settings. Dex reduced inflammatory cell infiltration and downregulated the expression of matrix-degrading enzymes to a certain extent in our experimental setting. Nevertheless, no significant improvements in joint range of motion or reduction in capsular fibrosis thickness were observed. These findings indicate that merely suppressing inflammation without restoring defective cellular energy metabolism may not effectively reverse capsular fibrosis in this rat model. Under the present study's dosage, AD-Mito-NPs exerted preferable therapeutic performance in both joint functional recovery and histological improvement via its dual regulatory mechanism.

In terms of clinical translation, ADSCs are widely accessible and can be harvested through minimally invasive liposuction [28,54], which meets the clinical needs of autologous transplantation while avoiding ethical concerns and immune rejection. The manufacturing process, involving cell culture, membrane and mitochondrial isolation, and extrusion assembly, is relatively well-defined and suitable for large-scale production. The treatment method is local injection, which aligns with the commonly used intra-articular injection in clinical practice and is easily accepted by both doctors and patients. One of the limitations of our study is that we currently lack relevant pharmacokinetic data and data reflecting the *in vivo* clearance profile following intra-articular administration. In addition, our single-cell sequencing data were obtained from human samples. Future studies may perform single-cell sequencing using rat glenohumeral joint capsule specimens. Moreover, follow-up research needs to establish prolonged post-treatment observation and perform additional characterizations, such as dose-response profiling, long-term biosafety assessment, pain behavioral assays and disease recurrence monitoring.

## 5 Conclusion

This study confirms that mitochondrial metabolic dysfunction plays a critical role in driving joint capsule fibrosis in adhesive capsulitis. We have developed AD-Mito-NPs and demonstrated their effectiveness in reversing fibrotic pathology and restoring joint function through a multi-mechanistic approach. It may serve as a candidate platform for the targeted therapy of adhesive capsulitis and warrants further investigation for potential clinical translation.

## CRediT authorship contribution statement

Yiming Zhu, Supervision, Conceptualization, Investigation and Writing—review and editing; Zeyu Wang, Conceptualization, Investigation and Writing—review and editing; Ziqi Huo, Investigation; Dan Zhang, Investigation; Yang Zhao, Investigation; Jianhao Xie, Investigation; Hao Li, Investigation; Tianyuan Zhao, Investigation; Pinxue Li, Investigation; Zhi Wang, Investigation; Chunyan Jiang, Supervision and Conceptualization; Xu Li, Conceptualization, Investigation, Writing—original draft preparation and Writing—review and editing.

## Ethics declaration

Written informed consent to take part in the study and to publish the article has been obtained from all participants or their legal representatives. The privacy rights of participants have been observed.

This study included organ or tissue donors. This study includes human biological material and consent was obtained by donors, or their next of kin or legal representatives, for use in this study and for publication of the article. The samples used in this research were not sourced from executed prisoners or prisoners of conscience.

This study was performed in compliance with relevant laws, regulatory frameworks and guidelines where the research took place. This study was approved by the Ethics Committee(seal)of Beijing Jisbuitan Hospital, Capital Medical University. (Approval No. K2026-290-00)

This study was conducted in accordance with the ARRIVE (Animal Research: Reporting of In Vivo Experiments) guidelines. This study was approved by the Animal Ethic Committee of Beijing Jishuitan Hospital. (Approval No. 2024-08-05)

## Availability of data and materials

The data supporting the findings of this study are available from the corresponding authors upon reasonable request.

## Declaration of generative AI and AI-assisted technologies in the writing process

During the preparation of this work the authors used Doubao AI in order to proofread grammatical errors and improve language. After using this tool, the authors reviewed and edited the content as needed and took full responsibility for the content of the publication.

## Funding

This study was supported by the 10.13039/501100001809National Natural Science Foundation of China (82402804), R&D Program of 10.13039/501100003213Beijing Municipal Education Commission (1240030172), Beijing Jishuitan Research Funding (JSTYC202403; ZR-2024; QN-2024-06), and Beijing High-Level Innovation and Entrepreneurship Talent Support Program-Healthcare Platform-Leading Talent Projects (G202511033).

## Declaration of competing interests

The authors declare that they have no known competing financial interests or personal relationships that could have appeared to influence the work reported in this paper.
