# Supplementary material for: ADSC-derived mitochondrial nanovesicles transplantation alleviates capsular fibrosis and inflammation and improves joint mobility in a rat model of adhesive capsulitis
Source: J Orthop Translat. 2026 Aug 22;60:101195. doi: 10.1016/j.jot.2026.101195 (PMC13524645; doi:10.1016/j.jot.2026.101195)
Supplement: Supplementary file 1 [file mmc1.docx]

**Fig. S1.** Expression of ATP synthesis and oxidative phosphorylation-related genes. (A) Expression of marker genes projected on UMAP. (B) Violin plots illustrating the expression of ATP synthesis and oxidative phosphorylation-related genes in fibroblasts, macrophages and overall cell populations.

**Fig. S2.** Expression of genes associated with ATP synthesis and oxidative phosphorylation in fibroblasts. (A) Volcano plot showing differentially expressed genes in fibroblasts between adhesive capsulitis tissues and normal tissues. (B) Dynamic changes in the proportional distribution of fibroblast subpopulations. (C) Violin plots illustrating the expression levels of marker genes in fibroblast subclusters. (D) Violin plots illustrating the expression levels of genes involved in ATP synthesis and oxidative phosphorylation in fibroblast subclusters.

**Fig. S3.** Flow cytometric quantification of CD90 (FITC)- and MitoTracker (PE)-labeled AD-Mito-NPs.

**Fig. S4.** Representative confocal images and quantification of intracellular ROS levels detected by DCFH-DA fluorescence. Green: DCFH-DA (ROS); Blue: Hoechst (nuclei). Scale bar, 50μm.

**Fig. S5.** H&E staining of major organs after intra-articular injection of AD-Mito-NPs.
